# Supplementary material for: Reconciling Mining with the Conservation of Cave Biodiversity: A Quantitative Baseline to Help Establish Conservation Priorities
Source: PLoS One. 2016 Dec 20;11(12):e0168348. doi: 10.1371/journal.pone.0168348 (PMC5173368; doi:10.1371/journal.pone.0168348)
Supplement: S1 Dataset — (ZIP) [file pone.0168348.s002.zip › Taxa/Serra Norte/SN_2013/N4E/Lista N4E-58.pdf]

CAVIDADE N4E-0058

Classe	Ordem	Fam/Outros	Gên/Outros	Espécie	S-U	U-S	Zon.
Arachnida	Araneae	Pholcidae	*Leptopholcus*	sp.1	X		E
Arachnida	Araneae	Pholcidae	*Mesabolivar*	sp.1	X		E
Arachnida	Araneae	Pholcidae		jovem		X	E
Arachnida	Araneae	Salticidae		jovem		X	E
Arachnida	Araneae	Scytodidae	*Scytodes*	*eleonorae*		2	E
Arachnida	Araneae	Uloboridae	*Uloborus*	sp.1		X	E
Arachnida	Opiliones	Stygnidae	*Protimesius*	*gracilis*		1	E
Arachnida	Pseudoscorpiones	Chernetidae	*Spelaeochernes*	sp.1		X	E
Entognatha	Collembola	Paronellidae		sp.1		X	E
Insecta	Blattodea			jovem		1	E
Insecta	Coleoptera			jovem		X	E
Insecta	Diptera	Cecidomyiidae	Cecidomyiinae	sp.		X	E
Insecta	Diptera	Chloropidae		sp.		X	E
Insecta	Diptera	Dolichopodidae		sp.	X		E
Insecta	Diptera	Tipulidae	Tipulinae	sp.		X	E
Insecta	Heteroptera	Nabidae	*Arachnocoris*	sp.1		X	E
Insecta	Heteroptera	Ochteridae		sp.		X	E
Insecta	Heteroptera	Reduviidae	Emesinae	sp.		1	E
Insecta	Hymenoptera	Bethylidae		sp.3		X	E
Insecta	Hymenoptera	Formicidae	*Pheidole*	sp.2		X	E
Insecta	Hymenoptera	Formicidae	*Pheidole*	sp.3		X	E
Insecta	Lepidoptera	Noctuoidea		sp.5	1		E
Insecta	Orthoptera	Phalangopsidae	*Paraclodes*	sp.		2	E
Insecta	Psocoptera	Psyllipsocidae	*Psyllipsocus*	sp.3		X	E
Malacostraca	Isopoda	Dubioniscidae		sp.1	X		E
